# Conversion of Gastrointestinal Somatostatin-Expressing D Cells Into Insulin-Producing Beta-Like Cells Upon *Pax4* Misexpression

**DOI:** 10.3389/fendo.2022.861922

**Published:** 2022-04-29

**Authors:** Anna Garrido-Utrilla, Chaïma Ayachi, Marika Elsa Friano, Josipa Atlija, Shruti Balaji, Tiziana Napolitano, Serena Silvano, Noémie Druelle, Patrick Collombat

**Affiliations:** ^1^ Université Côte d’Azur, Centre National de la Recherche Scientifique (CNRS), Institut National de la Santé Et de la Recherche Médicale (INSERM), Institut de Biologie Valrose (iBV), Nice, France; ^2^ Department of Cryopreservation, Distribution, Typing and Animal Archiving, Centre National de la Recherche Scientifique-Unité d'Appui à la Recherche (CNRS-UAR) 44 Typage et Archivage d’Animaux Modèles (TAAM), Orléans, France; ^3^ PlantaCorp Gesellschaft mit beschränkter Haftung (GmbH), Hamburg, Germany; ^4^ Columbia University College of Physicians & Surgeons, Department of Medicine, New York, NY, United States

**Keywords:** diabetes, somatostatin, pax4, gastrointestinal tract, mouse

## Abstract

Type 1 diabetes results from the autoimmune-mediated loss of insulin-producing beta-cells. Accordingly, important research efforts aim at regenerating these lost beta-cells by converting pre-existing endogenous cells. Following up on previous results demonstrating the conversion of pancreatic somatostatin delta-cells into beta-like cells upon *Pax4* misexpression and acknowledging that somatostatin-expressing cells are highly represented in the gastrointestinal tract, one could wonder whether this *Pax4*-mediated conversion could also occur in the GI tract. We made use of transgenic mice misexpressing *Pax4* in somatostatin cells (SSTCrePOE) to evaluate a putative *Pax4*-mediated D-to-beta-like cell conversion. Additionally, we implemented an *ex vivo* approach based on mice-derived gut organoids to assess the functionality of these neo-generated beta-like cells. Our results outlined the presence of insulin^+^ cells expressing several beta-cell markers in gastrointestinal tissues of SSTCrePOE animals. Further, using lineage tracing, we established that these cells arose from D cells. Lastly, functional tests on mice-derived gut organoids established the ability of neo-generated beta-like cells to release insulin upon stimulation. From this study, we conclude that the misexpression of *Pax4* in D cells appears sufficient to convert these into functional beta-like cells, thus opening new research avenues in the context of diabetes research.

**Graphical Abstract:**
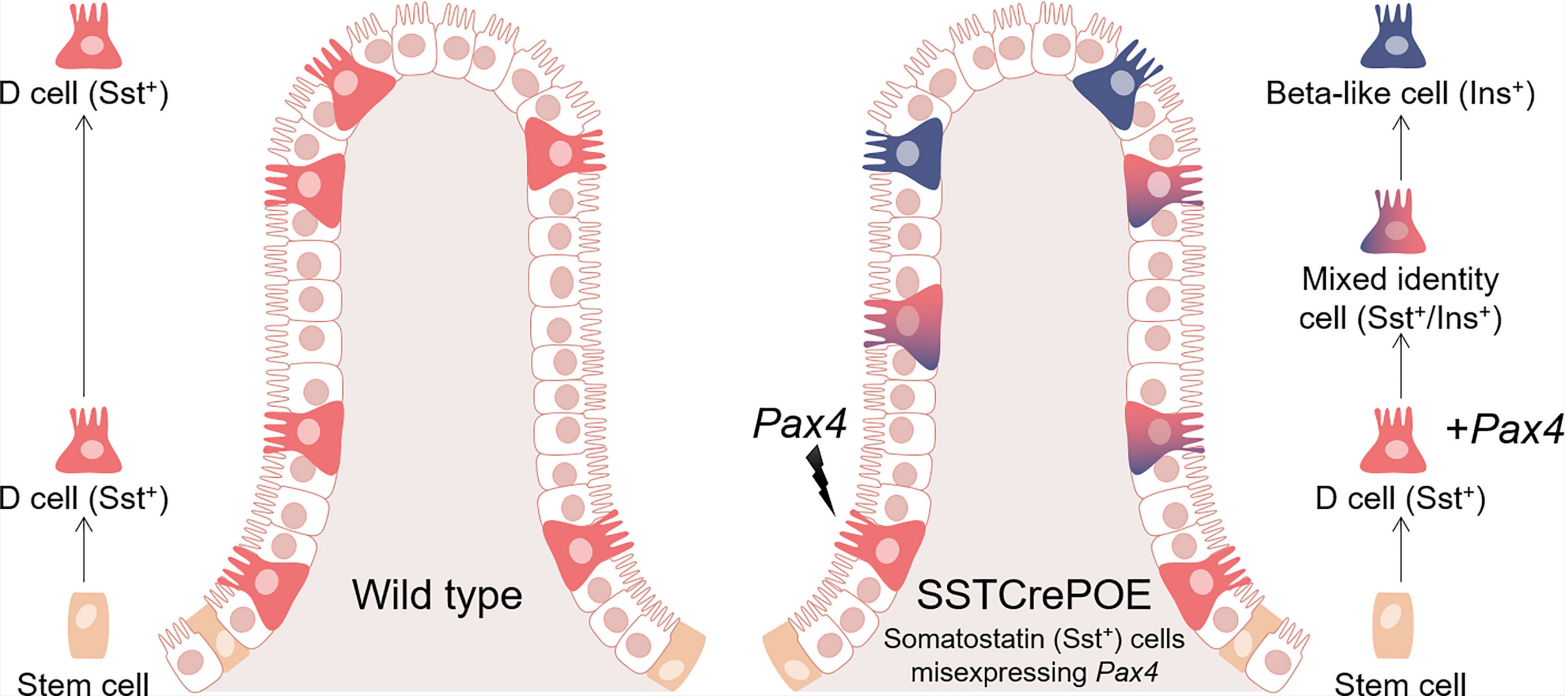
Schematic representation of wild-type (left) and SSTCrePOE (right) gastrointestinal tracts. D cells normally arise in the crypts and progress towards the tip of the villi (left). In SSTCrePOE mice (right), while a similar cell migration is detected, the misexpression of Pax4 in D cells results in their gradual conversion into beta-like cells.

## Introduction

Type 1 Diabetes Mellitus corresponds a metabolic disease resulting from the autoimmune-mediated loss of insulin-producing beta-cells, which leads to a chronic hyperglycaemia (1–4). Despite currently available therapies (mostly based on insulin supplementation), type 1 diabetic patients still display a significantly altered life quality and a shortened life expectancy. Therefore, alternative therapies are required. Towards this goal, numerous attempts aim at regenerating/replacing the pool of insulin-producing cells making use of stem cells (5, 6), pluripotent cells (7–19), or by reprogramming somatic cells (20–33).

Interestingly, most of these approaches rely on the modulation of developmental genes, a majority encoding for transcription factors. Among these, *Pax4* plays a crucial role in the differential endocrine cell allocation during pancreas morphogenesis (34–36). Accordingly, mice depleted of *Pax4* are hyperglycaemic and die shortly postpartum due to a lack of insulin-producing beta- and somatostatin-secreting delta-cells (37–39). Conversely, the number of glucagon-expressing alpha-cells is proportionally increased in *Pax4* mutant mice. These results indicate that, during development, *Pax4* promotes the allocation to the beta- and delta-cell lineages at expense of the alpha- (but also epsilon-) cell fate(s) (40).

Importantly, mice misexpressing *Pax4* in alpha-cells were found able to recover from several cycles of chemically-induced diabetes as a result of beta-like cell neogenesis (20, 21, 41, 42). Specifically, it was demonstrated that the ectopic expression of *Pax4* in pancreatic glucagon-producing alpha-cell was sufficient to induce their neogenesis and their subsequent conversion into insulin-producing beta-like cells (20, 22).

Furthermore, it was recently shown that in the pancreas of mice ectopically expressing *Pax4* in somatostatin cells, larger and increased numbers of islets were observed when compared with matched controls, such difference progressively increasing with age. This increment in islets size was correlated with an increase in the number of insulin^+^ cells (41). The results obtained demonstrated that somatostatin cells were continuously regenerated and converted into insulin-expressing cells. Further analysis confirmed that these beta-like cells were fully functional and could partially reverse chemical-induced diabetes. Altogether, these results indicated that the neo-generated insulin-expressing cells were phenotypically and functionally true beta-cells. It is worth noting that the vast majority of circulating somatostatin originates from the D cells located along the GI tract (43, 44), accounting for 65% of the total amount of the hormone, whereas the central nervous system is responsible for producing around 25% of it and the pancreas releases approximately 5% of the total somatostatin (44, 45). D cells are found scattered into the gastrointestinal mucosa, being highly represented in the duodenum and in the stomach (43, 44, 46–48). In fact, D cells, which produce somatostatin and are scattered along the gut epithelium, produce approximately 65% of the whole-body circulating hormone. One could therefore wonder whether the *Pax4*-mediated conversion of somatostatin-producing cells into insulin-expressing cells could also occur in the gastrointestinal tract, an information of great importance for type 1 diabetes research.

## Material and Methods

### Mouse Strain: SSTCrePOE

A bi-transgenic mouse line, the SSTCrePOE mouse line (41), was generated by crossing two single transgenic mouse lines: SST-Cre (49) and Pax4-OE (20) which were created using classical pronuclear injection. This transgenic mouse model allows the ectopic and constitutive expression of Pax4 in somatostatin-producing cells.

### Animal Procedures and Ethics Statement

Experiments were performed on transgenic and control mice, control mice corresponding to SST-Cre-negative and Pax4-OE littermates. Transgenic and control animals were found to be viable, healthy, and fertile, with no premature death observed. All animals were housed on a standard 12:12-h light/dark cycle, with standard diet food and water ad libitum. Animal protocols were reviewed and approved by an institutional ethics committee (Ciepal-Azur) at the University of Nice, and all colonies were maintained following European animal research guidelines. This project received approval from our local ethics committee.

### Tissue Immunofluorescence Assessment

Tissues were fixed in 4% paraformaldehyde for 30 min at 4°C, embedded in paraffin and 5μm sections applied to glass slides. These sections were assayed using DAPI as counterstain. The primary antibodies used were guinea pig anti-insulin (1/500; DAKO), rat anti-somatostatin (1/250; Millipore), goat anti-somatostatin (1/500; Santa Cruz), rabbit anti-PC1/3 (1/500; Millipore), rabbit anti-NeuroD1 (1/500; Millipore), rat anti-C-peptide (1/500; Phoenix pharmaceuticals), rabbit anti-Hhex (1/50; R&D. The secondary antibodies (1/1,000; Invitrogen and Jackson ImmunoResearch) used were Alexa Fluor 488, 594, and 647 and Cy3 and Cy5. Images were collected at room temperature on an AxioImagerZ.1 upright microscope (Zeiss) equipped with a Zeiss 10× Plan-Neofluar dry NA 0.3 and/or Zeiss 20× Plan-Apochromat dry NA 0.8 and/or Zeiss 20× Plan-Neofluar dry NA 0.5, a DAPI filter (excitation [Ex] 365/12 nm, dichroic mirror [DM] 395 nm, emission [Em] 445/50), a GFP filter (Ex 470/40 nm, DM 495 nm, and Em 525/50 nm), a rhodamine filter (Ex 546/12 nm, DM 560 nm, and Em 608/65 nm), a Texas red filter (Ex 560/40 nm, DM 582 nm, and Em 630/785 nm), and/or a Cy5 filter (Ex 640/30 nm, DM 660 nm, Em 690/50 nm).

### β-Galactosidase-Based Lineage Tracing Experiments

Tissues were isolated and fixed for 30 min at 4°C in a solution containing 1% formaldehyde, 0.2% glutaraldehyde, and 0.02% NP-40 and washed three times in cold PBS. The tissues were dehydrated in PBS-25% sucrose overnight at 4°C. Tissues were washed 2h in Jung freezing medium (Leica Biosystems) and embedded in freezing medium on dry ice. 10μm sections were applied to glass slides, dried, and washed three times in PBS. After 45min blocking in PBS-FCS 5%, sections were incubated with a rabbit anti-β-galactosidase primary antibody (1/8,000; MP-Cappel).

### RNA Analyses

Mice were euthanized by cervical dislocation and a piece of tissue was isolated and placed in cold RNA later solution (Thermo Fisher Scientific) for 48 h at 4°C. RNA was isolated (RNAeasy; QIAGEN), and cDNA synthesis (SuperScript choice system; *Invitro*gen) was performed according to the manufacturer’s instructions.

### Gene Expression Analyses

Quantitative RT–PCR was performed using validated primers (QIAGEN) and the QuantiTect SYBR Green RT-PCR kit (QIAGEN) following the manufacturer’s instructions. PCR reactions and detection were performed on a Mastercycler ep realplex cycler using GAPDH as an internal control for normalization purposes.

### Glucose Tolerance Tests and Blood Glucose Level Measurements

For challenge purposes, animals were fasted for 6-8h and injected intraperitoneally with 2g/kg body weight glucose. Blood glucose levels were measured at the indicated time points post injection with a ONE TOUCH Vita glucometer (Life Scan, Inc.).

### Organoid Generation

Organoids were generated following the protocols described in literature (50, 51). Briefly, animals were sacrificed by cervical dislocation or by decapitation and duodenum was harvested and the villi were scraped and removed. The duodenum was incubated in 2mM EDTA-DPBS for 1h at 4°C. The supernatant was removed, and 10ml of 10% FBS-DPBS were added and the samples were vigorously shaken. The supernatant was collected and passed through a 70μm strainer. The strainer flow-through was centrifuged at 300g for 5min so the crypts were spun down. The number of crypts was calculated using a Neubauer chamber and the crypts were cultured at a range of 5000 crypts per well. To proceed with plating the crypts, the sample was spun down at 200g for 2min, the supernatant was aspirated, and the pellet was resuspended in 50% Matrigel-Growth medium. 50μl of the solution was plated and placed into the incubator at 37°C and 5% of CO_2_ for 2min in order to allow the Matrigel to solidify and after, 500μl of growth medium was added into each well. The organoids were fed every 3 days and split in a ratio 1:6 every 10 days.

### Organoid Immunofluorescence

Organoids were assayed by immunofluorescence following a previously protocol (51). Briefly, medium was aspirated from the wells. PFA 4%, was added into the wells and incubated for 20min at RT. Once the incubation finished, the fixative was removed and DPBS was added. All the well content was transferred in a 15ml falcon tube and centrifuged for 5min at 800rpm. The supernatant was discarded and a permeabilization buffer, which is a solution of 0.2% Triton in DPBS, was poured into the organoids pellet and incubated for 30min at 4°C on a rocking plate. Once the incubation was completed, samples were centrifuged 5min at 800rpm and the supernatant was discarded. A blocking step was performed by adding during 30min at RT and on a rocking plate 10% FBS-DPBS. Then, the normal immunofluorescence steps were performed (primary antibody ON at 4°C and secondary antibody for 45min at RT).

### Glucose-Stimulated Insulin Secretion (GSIS)

The medium was aspirated and 1ml of fresh prepared 2.8mM Glucose-Krebs Ringer Buffer was added. The organoids were incubated for 30min at 37°C. The medium was aspirated and 500μl of new 2.8mM Glucose-Krebs Ringer Buffer added. The samples underwent another incubation of 30min at 37°C. The medium was collected and snap-freeze to determine the basal insulin concentration. Afterwards, 500μl of 16.8mM Glucose-Krebs Ringer Buffer was added into the wells and samples were incubated. After a 30-minute incubation, the medium was collected and snap freeze in liquid nitrogen. This medium will serve to assess insulin release upon glucose stimulation. Finally, RIPA buffer was added into each well pipetted back and forth and well content was collected into Eppendorf’s. The tubes were centrifuged for 20min at 4°C at 10000rpm and supernatant was collected and frozen to perform a BCA assay. The samples were analysed by using the Mouse Ultrasensitive Elisa kit (Mercodia).

### Data Analyses

All values are depicted as mean ± SEM of data from at least three animals. Data were analysed using GraphPrism 7 software. Normality was tested using a D’Agostino–Pearson omnibus normality test, and appropriate statistical tests were performed. Results are considered significant if P < 0.001 (***), P < 0.01 (**), and P < 0.05 (*).

## Results

### *Pax4* Misexpression in D Cells Results in Their Conversion Into Insulin-Expressing Cells

**Figure 1:**
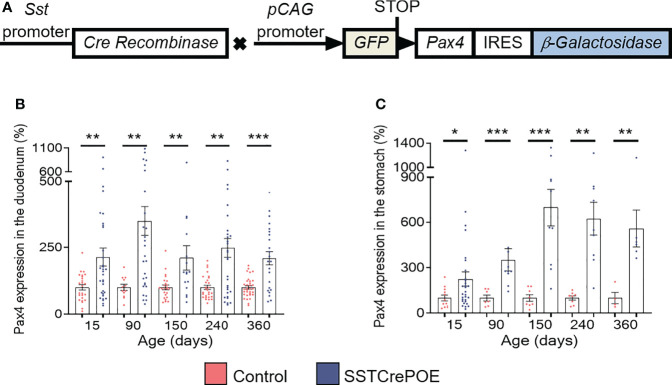
Generation and validation of the SSTCrePOE mouse line. **(A)** SSTCrePOE mouse line was generated by crossing two single transgenic mouse lines: SST-Cre and Pax4-OE which were created using classical pronuclear injection. SSTCrePOE animals ubiquitously express Green Fluorescent Protein (GFP). However, in somatostatin-expressing cells, the Cre recombinase promotes the loss of the *LoxP-GFP-STOP-LoxP* cassette and thus, *Pax4* and *β-galactosidase* misexpression. **(B, C)** Comparison of *Pax4* expression levels in the duodenum **(B)** and stomach **(C)** of control (red) and SSTCrePOE (blue) mice (4 < n < 33). Note the massive increase in *Pax4* expression both in SSTCrePOE duodenum and stomach. *P < 0.05, **P < 0.01, ***P < 0.001.

To investigate the consequences of *Pax4* misexpression in gastrointestinal D cells, we made use of the SSTCrePOE transgenic mouse model allowing the ectopic and constitutive expression of *Pax4* in somatostatin-producing cells (**Figure 1A**). Such double transgenic animals harboured a first transgene (SST-Cre) composed of the *Somatostatin (Sst)* promoter driving the expression of the Cre recombinase and a second (Pax4-OE) encompassing the ubiquitously expressed *pCAG* promoter upstream of a *LoxP-GFP-STOP-LoxP* cassette followed by a *Pax4-IRES-β-Galactosidase* cassette (20, 41). Thus, SSTCrePOE animals both allowed *Pax4* misexpression in somatostatin^+^ cells and the tracing of their lineage. Control mice were SST-Cre-negative Pax4-OE littermates. Importantly, a significant increase in *Pax4* expression (up to 599%) was detected both in the duodenum and the stomach of SSTCrePOE animals when compared to age matched controls (**Figures 1B, C**). Unfortunately, due to the lack of working anti-Pax4 antibodies, the detection of Pax4 at the protein level could not be performed. However, the exclusive misexpression of Pax4 in somatostatin^+^ cells had been previously validated in SSTCrePOE pancreata (41).

**Figure 2:**
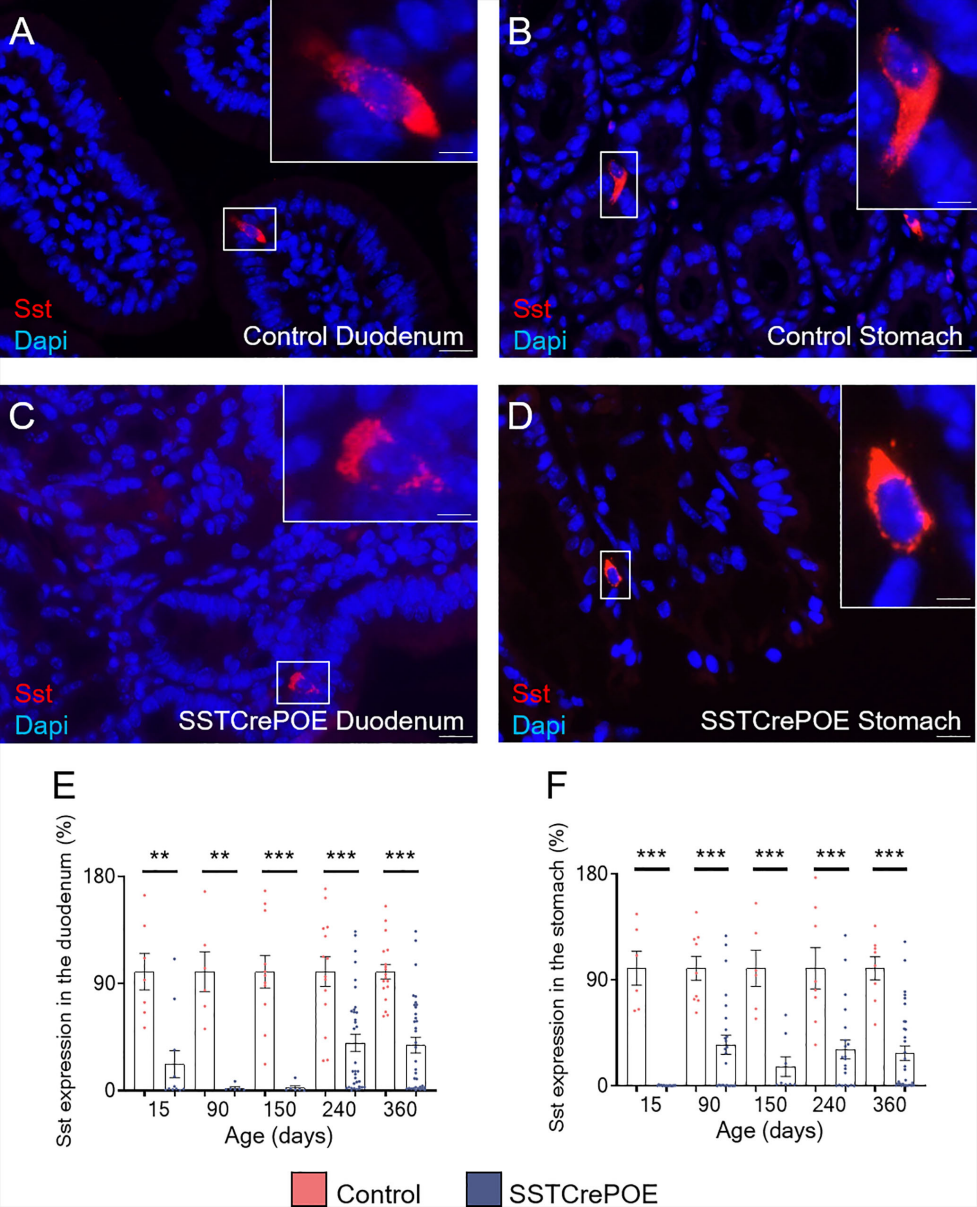
The misexpression of *Pax4* in D cells induces their loss both in duodenum and stomach. **(A–D)** Representative photographs of somatostatin-expressing D cells detected in the duodenum **(A, C)** and stomach **(B, D)** of control **(A, B)** and transgenic **(C, D)** samples. The displayed photographs correspond to 90-day-old animals, similar results being observed at 15, 150, 240 and 360 days of age. Scale bar, 20μm, insets 5 μm. **(E, F)** Comparison of *somatostatin* expression levels in the duodenum **(E)** and stomach **(F)** of control (red) and SSTCrePOE (blue) mice (6 < n < 35). Note the dramatic decrease in *somatostatin* expression in SSTCrePOE duodenum and stomach and the no statistical difference detect between ages of SSTCrePOE samples. **P < 0.01, ***P < 0.001.

D cells are generally found scattered along the gut epithelium throughout life. Accordingly, using different approaches, we confirmed the presence of D cells both in the stomach and the duodenum. Specifically, somatostatin^+^ cells were detected by immunofluorescence in control duodenum and in the stomach at all ages tested, that are 15, 90, 150, 240 and 360 days of age (**Figures 2A, B**). Importantly, a strong reduction in the content in somatostatin^+^ cells was evidenced in SSTCrePOE animals at all ages tested, although few cells could still be detected (**Figures 2C, D**). Due to the difficulties to quantify scattered somatostatin-expressing cells in organs as large as the duodenum and the stomach, we resorted to qRT-PCR analyses. These confirmed our observations with a massive decrease in *somatostatin* transcript contents in SSTCrePOE transgenic samples (up to 97% depending on the (st)age analysed) when compared to control littermates (**Figures 2E, F**). In line with this strong reduction in somatostatin expression, somatostatin circulating levels were also found reduced by 35% when compared to controls (**Supplementary Figure 1A**), while no significant difference was observed in circulating levels of IL-1beta and TNF-alpha proinflammatory cytokines (**Supplementary Figures 1B, C**), suggesting that the gastrointestinal barrier function of SSTCrePOE animals remained unaltered.

**Figure 3:**
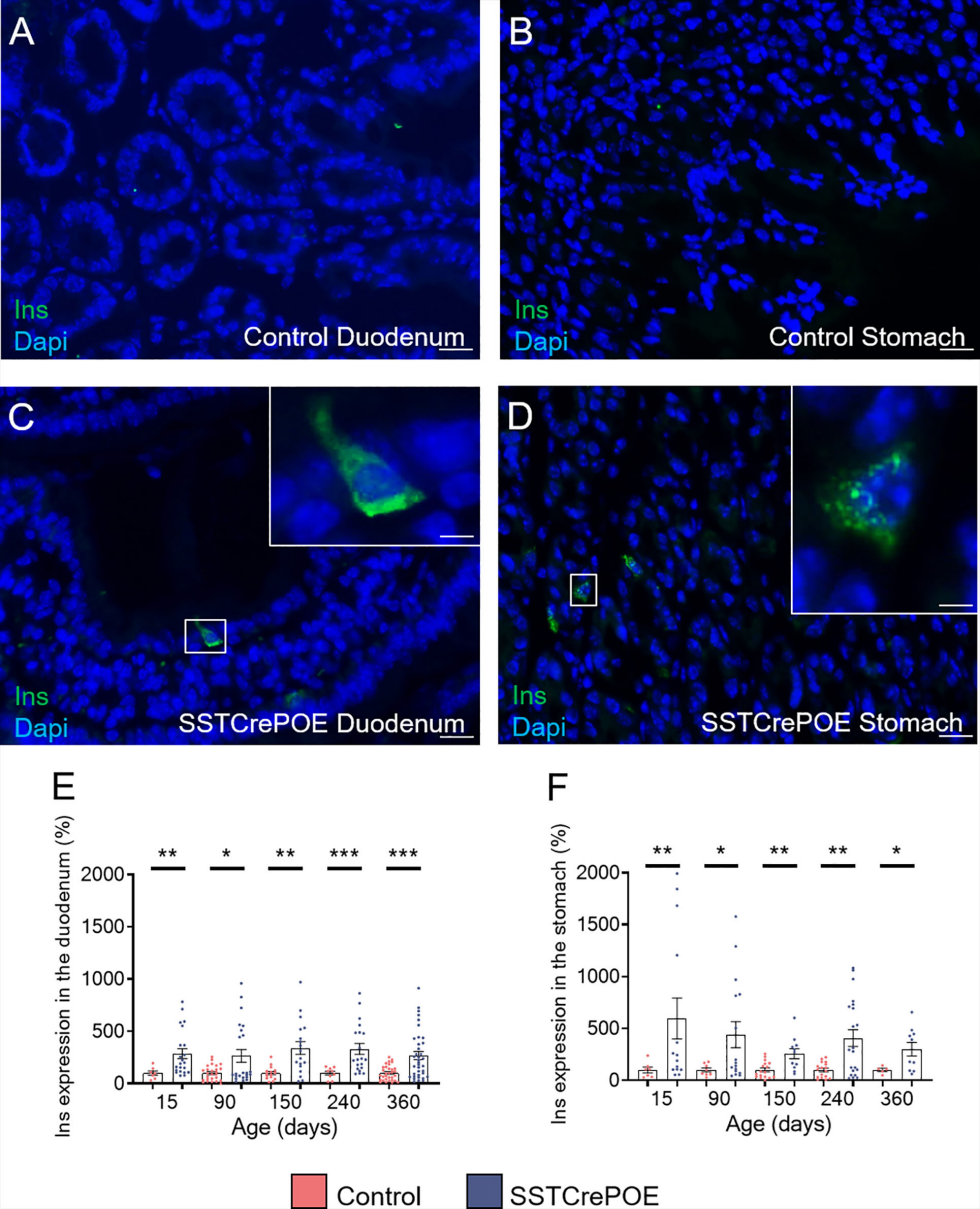
Detection of insulin-expressing cells in SSTCrePOE animals. **(A–D)** Representative photographs of immunohistochemical analyses focusing on insulin in the duodenum **(A, C)** and in the stomach **(B, D)** of control **(A, B)** and transgenic **(C, D)** samples. Insulin-producing cells are clearly detected solely in SSTCrePOE tissues. The displayed photographs correspond to 150-day-old animals, similar results being observed at 15, 90, 240 and 360 days of age. Scale bar, 20μm, insets 5 μm. **(E, F)** Expression level analyses confirmed a massive increase in *insulin* transcript contents both in SSTCrePOE duodenum **(E)** and stomach **(F)** when compared to controls (5 < n < 37). *P < 0.05, **P < 0.01, ***P < 0.001.

Aiming to determine whether the reduction in D cell numbers observed in transgenic animals could be explained by the acquisition of an alternative identity, as previously reported in the pancreas (41), further analyses were undertaken with a focus on insulin. It is important to note that insulin^+^ cells are normally never detected in the gastrointestinal tract of wild-type mice (**Figures 3A, B**). However, and most surprisingly, insulin^+^ cells were clearly observed in SSTCrePOE transgenic duodenum and stomach (**Figures 3C, D**). Accordingly, gene expression studies confirmed a dramatic increase in *insulin* transcript contents (up to 495%) at all (st)ages tested both in the duodenum and stomach of SSTCrePOE mice when compared to control samples (**Figures 3E, F**).

**Figure 4:**
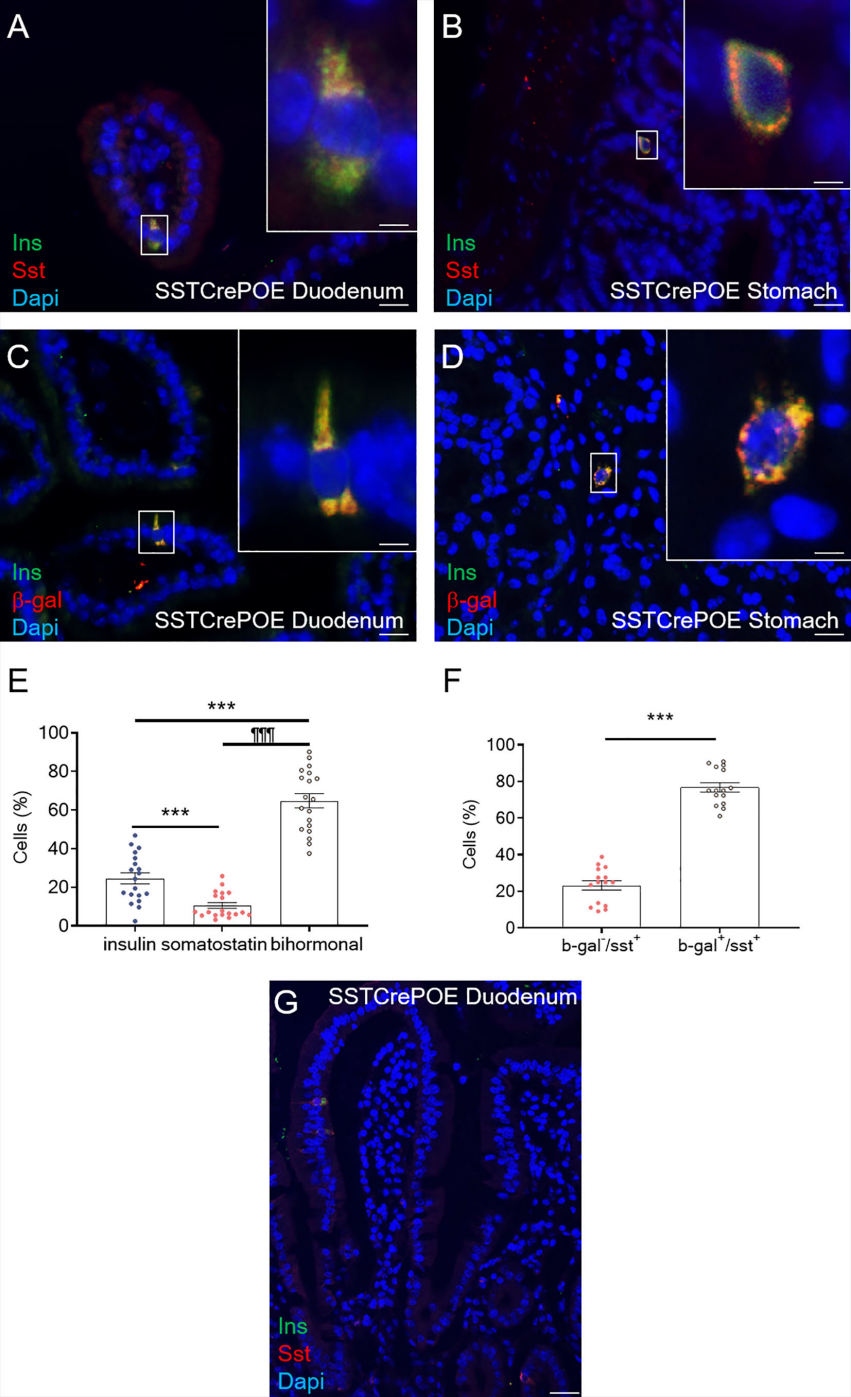
Conversion of D cells into insulin-expressing cells upon *Pax4* misexpression. **(A, B)** Detection in SSTCrePOE tissues of bi-hormonal cells expressing both somatostatin and insulin. **(C, D)** Representative photographs of the duodenum **(C)** and stomach **(D)** from SSTCrePOE animals stained for insulin and β-galactosidase, demonstrating a conversion of somatostatin^+^ cells into insulin^+^ cells. Note that all insulin^+^ cells are labelled with β-galactosidase. The displayed photographs correspond to 360-day-old animals, similar results being observed at 15, 90, 150 and 240 days of age. Scale bar, 20μm, insets 5 μm. **(E)** Quantification of cells expressing insulin, somatostatin and both hormones in 3 duodenal sections of 4 independent SSTCrePOE mice from different ages (15, 90, 150, 240 and 360 days of age). Importantly, 10.5% of D cells retained their identity by solely expressing somatostatin, 65% of cells were found to coexpress both two hormones (insulin and somatostatin), and 24.5% of cells acquired a single-hormone insulin-expressing cell phenotype. **(F)** Assessment of Cre recombinase efficiency in SSTCrePOE animals. 3 duodenal sections of 3 independent SSTCrePOE animals from different ages (15, 90, 150, 240 and 360 days of age) were assayed for somatostatin and beta-galactosidase. The results obtained showed that 76.5% of D cells were labelled with β-galactosidase and that 100% of insulin^+^ cells were β-galactosidase^+^ (**C, D** and data not shown). **(G)** Representative photograph outlining the presence of somatostatin^+^ cells closer to the crypts while insulin-expression is progressively detected closer to the tip of the villi, supporting the notion of a progressive conversion of somatostatin^+^ cells into insulin-expressing cells. ***P < 0.001, ^¶¶¶^P < 0.001.

Additional analyses were carried out to pinpoint the exact origin of gastrointestinal insulin-expressing cells. We first evaluated the main cell subtypes from the gastrointestinal mucosa (**Supplementary Figures 2A–E**) focusing on epithelial, Goblet, Paneth, Stem and Enteroendocrine cells. The results obtained showed that the insulin^+^ cells found in SSTCrePOE did express chromogranin A (ChgA), a marker of EECs, while being negative for Muc2, Lyz and Dcamkl1 markers of goblet, Paneth, and stem cell respectively. As gastrointestinal insulin-expressing cells were positive-labelled with ChgA (**Supplementary Figure 2E**), we assessed the expression of alternative gastrointestinal hormones: somatostatin, GLP1, GIP and gastrin (**Figures 4A, B**, **Supplementary Figures 2F–I** **).** Importantly, insulin^+^ cells were found negative for most of the assessed hormones (**Supplementary Figures 2F–I**) with the exception of somatostatin (**Figures 4A, B**). Indeed, 65% of insulin^+^ cells appeared to be bihormonal (Sst^+^/Ins^+^), while 24,5% of these were exclusively expressing this hormone, supporting the notion of a progressive/gradual conversion of somatostatin^+^ cells into insulin^+^ cells (**Figure 4E**). Importantly, somatostatin-expressing cells are mostly found located closer to the crypts whereas insulin-labelled cells are detected closer to the tip of the villi (**Figure 4G**), thus supporting the hypothesis that somatostatin^+^ cells are converted into insulin^+^ cells as they progress from the crypts to the top of the villi. Further investigations were then undertaken to determine the exact origin of gastrointestinal insulin-expressing cells. Towards this goal, we took advantage of the *β-galactosidase* reporter gene included in SSTCrePOE animals to trace the lineage of somatostatin^+^ cells (**Figure 4F**). Interestingly, the co-detection of both insulin and β-galactosidase (**Figures 4C, D**) demonstrated the conversion of D cells (permanently labelled with β-galactosidase upon *somatostatin* expression) into insulin^+^ cells. All insulin-expressing cells were thus found labelled with β-galactosidase. Together, these results suggest that the misexpression of the *Pax4* gene in D cells is sufficient to promote their reprogramming into insulin-producing cells.

### Gastrointestinal Insulin^+^ Cells Display a Beta-Like Cell Phenotype

**Figure 5:**
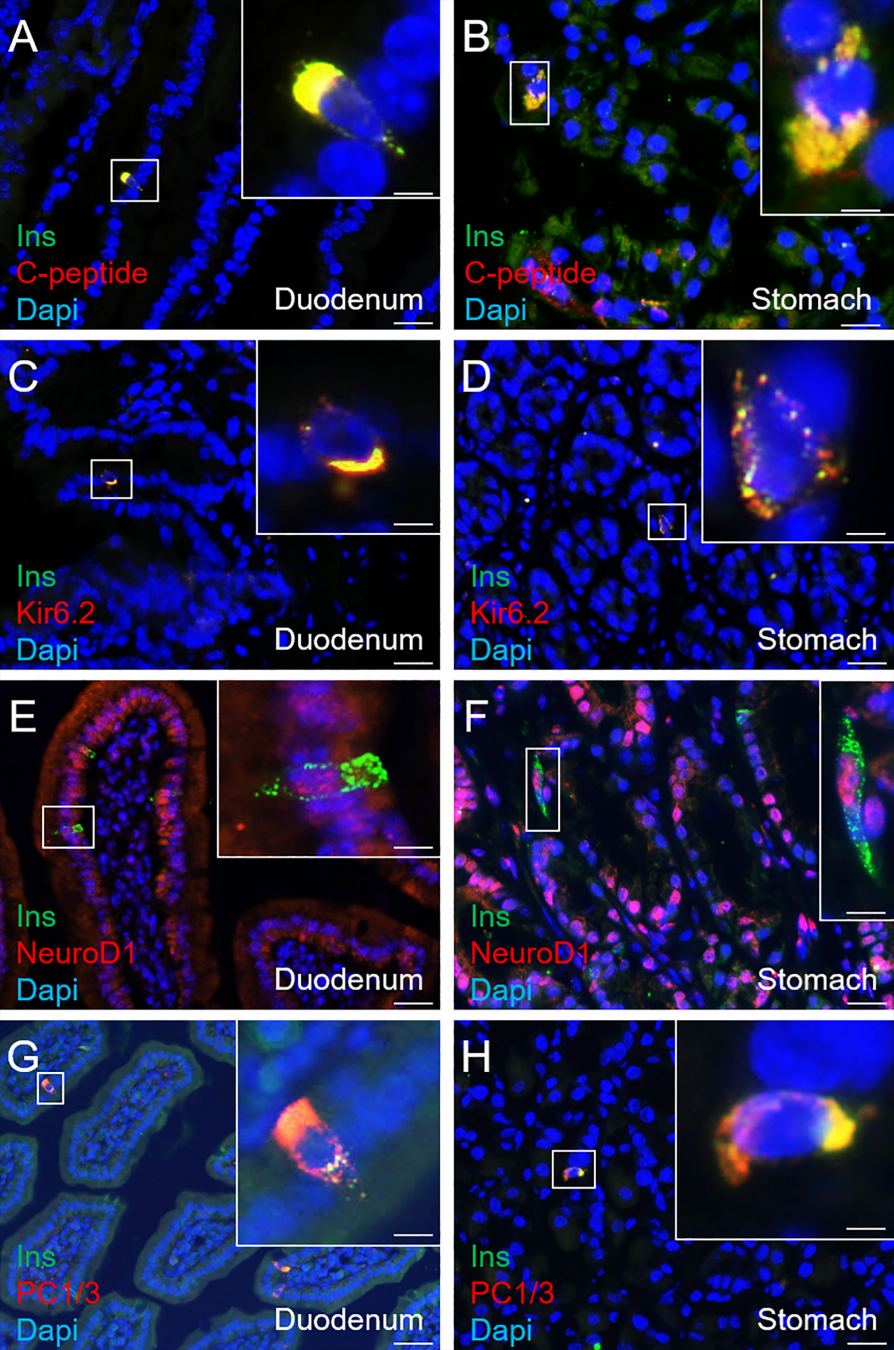
Immunohistochemical characterisation of insulin-expressing cells in SSTCrePOE animals. **(A–H)** Representative photographs of 90-, 150-, 240- and 360-day-old SSTCrePOE duodenum **(A, C, E, G)** and stomach **(B, D, F, H)** insulin-expressing cells assessed for the indicated *bona fide* beta-cell markers. Scale bar, 20μm, insets 5 μm. Noteworthy, insulin^+^ cells are found to co-express the assessed markers (C-peptide, Kir6.2, NeuroD1, PC1/3).

**Figure 6:**
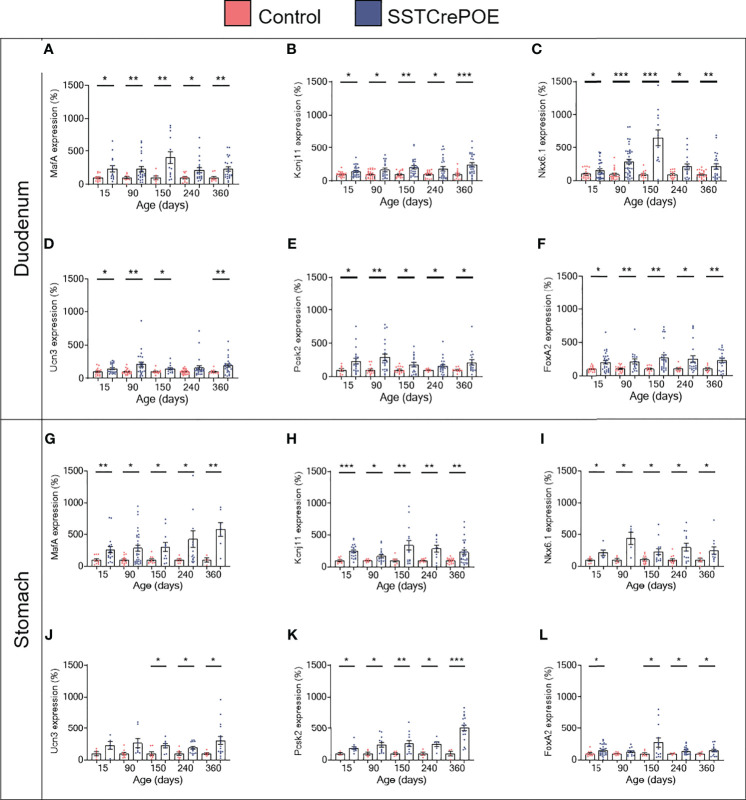
Gene expression analyses of SSTCrePOE duodenum and stomach. Comparison of the transcript levels between control (red) and transgenic (blue) duodenum (top panel) and stomach (bottom panel) samples at different ages focusing on beta-cell marker genes: **(A, G)** *MafA*, **(B, H)** *Kcnj11* encoding for Ki6.2, **(C, I)** *Nkx6.1*, **(D, J)** *Ucn3*, **(E, K)** *Pcsk2* encoding for PC2 and **(F, L)** *FoxA2* (3 < n < 40). **(D)** At 240-day-old the pvalue is 0,06. **(J)** At 15 and at 90-day-old pvalues are 0,1 and 0,06 respectively. **(L)** At 90-day-old the pvalue is 0,07. All beta-cell markers were found considerably increased on SSTCrePOE samples as compared to controls. *P < 0.05, **P < 0.01, ***P < 0.001.

Aiming to assess the identity of SSTCrePOE gastrointestinal insulin-expressing cells, a thorough characterisation was performed. Insulin^+^ cells from both SSTCrePOE duodenum and stomach were thus found to express *bona fide* beta-cell markers, such as C-peptide, Kir6.2 and NeuroD1 (**Figures 5A–F**). Moreover, gastrointestinal insulin^+^ cells were also positive for PC1/3, an enzyme not exclusively expressed in beta-cells but required for the maturation of insulin (**Figures 5G, H**). In addition to this immunohistochemical characterisation, a gene expression profile was established in gastrointestinal tissues by means of qRT-PCR. The expression levels of *MafA, Kcnj11* (encoding for Kir6.2)*, Nkx6.1, Ucn3, Pcsk2* (encoding for PC2) and *FoxA2* were all found increased in both the duodenum (up to 550%) and the stomach (up to 480%) of SSTCrePOE mice when compared to the control littermates (**Figure 6**). Taken together, these results suggest that, overall, gastrointestinal insulin-expressing cells display a beta-like cell identity.

**Figure 7:**
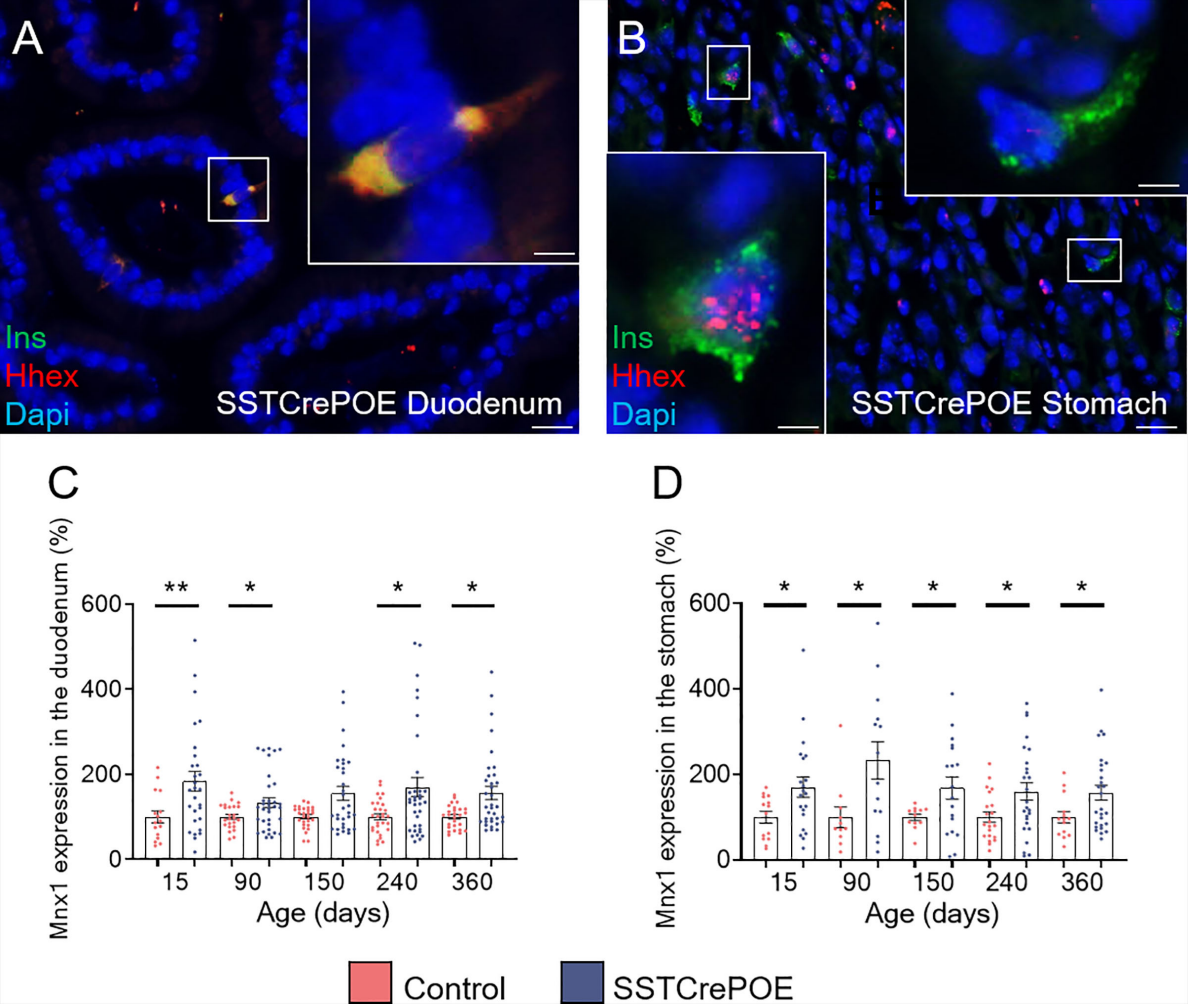
Assessment of the D cell identity loss in SSTCrePOE animals. **(A, B)** Representative photographs of SSTCrePOE duodenum **(A)** and stomach **(B)** revealing multiple localisations of Hhex, a well-characterise D cell marker. The displayed photographs correspond to 150-day-old animals, similar results being observed at 15, 90, 240 and 360 days of age. Scale bar, 20μm, insets 5 μm. **(C, D)** Assessment of *Mnx1* expression in the duodenum **(C)** a stomach **(D)** (12 < n < 35). **(C)** At 150-day-old the pvalue is 0,09. The significant increase in *Mnx1* transcript levels in SSTCrePOE samples suggest a role of *Mnx1* in the *Pax4*-mediated conversion of D cells into beta-like cells. *P < 0.05, **P < 0.01.

To determine whether somatostatin-expressing cells were fully losing their characteristics upon conversion into beta-like cells, the expression of the D cell-specific marker, Hhex, was examined (**Figures 7A, B**). Interestingly, the results obtained in SSTCrePOE animals outlined three subpopulations: (1) insulin^+^ cells retaining the expression of Hhex in their nuclei which could suggest a residual activity of the transcription factor (**Figure 7B**), (2) insulin^+^ cells displaying Hhex labelling in their cytoplasm indicative of a loss of the D cell identity by the translocation of Hhex into the cytoplasm (**Figure 7A**), and (3) insulin^+^ cells depleted of Hhex signal (**Figure 7B**). Such variability of Hhex expression could, yet again, be explained by the apparent progressive/gradual conversion process with the detection of cells at different stages, some of these thus displaying a mixed identity. Intriguingly, upon focusing on *Mnx1* (**Figures 7C, D**), which repression induces the inverse conversion (beta-to-delta) (52), a significantly increase in the expression of this gene was noted when comparing transgenic versus control gastrointestinal tissues, suggesting a putative role of *Mnx1* in the reprogramming of D cells into beta-like cells.

Taking into consideration our previous results, the functionality of gastrointestinal insulin^+^ cells was next explored. The results obtained with regards to controls animals outlined an improved glucose tolerance of SSTCrePOE mice with a lower glycemia peak and a faster return to euglycemia (**Supplementary Figure 3**). However, it is important to mention that one cannot directly correlate this improvement in glucose handling to the sole release of gastrointestinal insulin, as pancreatic delta-cells are also converted into insulin^+^ cells (41) and could therefore also contribute to this ameliorated glucose tolerance.

### Generation of an *Ex Vivo* Model

The aforementioned limitations of our *in vivo* model prompted us to turn to an *ex vivo* approach based on gut organoids. Gut organoids were thus generated from control and SSTCrePOE transgenic mice and thorough analyses were performed at day 90.

**Figure 8:**
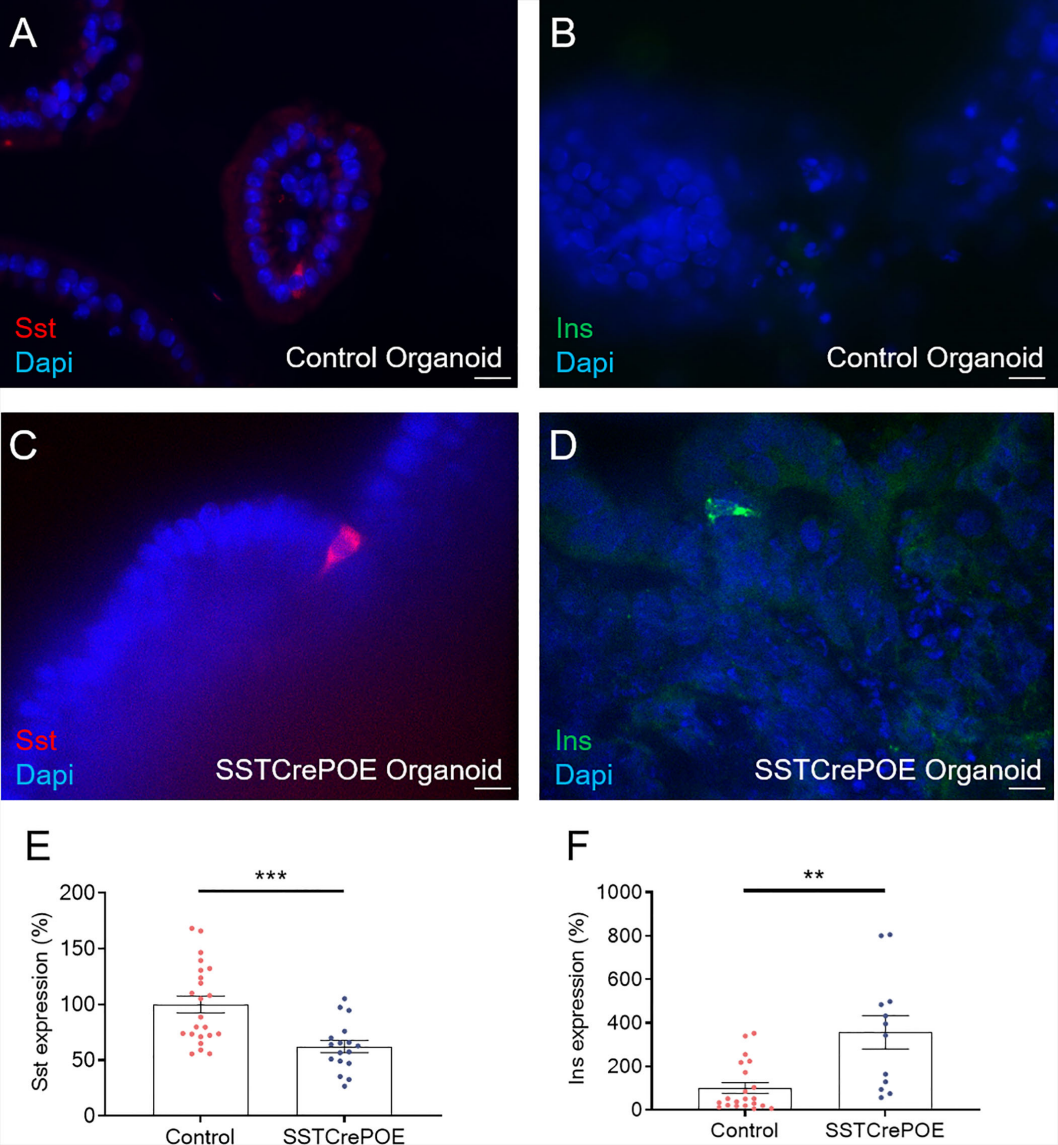
*Ex vivo* model validation. **(A–D)** Representative photographs of organoids assayed at day 90 by immunofluorescence: **(A, C)** D cells are readily detected in gut organoids derived from control **(A)** and transgenic **(C)** mice. **(B, D)** insulin-expressing cells are solely observed in SSTCrePOE gut organoids. Scale bar, 20μm. **(E, F)** Gene expression profiling demonstrate a **(E)** reduction of somatostatin transcript contents in transgenic gut organoids (17 < n < 23). **(F)** Conversely, insulin expression levels are massively increased in SSTCrePOE-derived organoids (12 < n < 21). **P < 0.01, ***P < 0.001.

**Figure 9:**
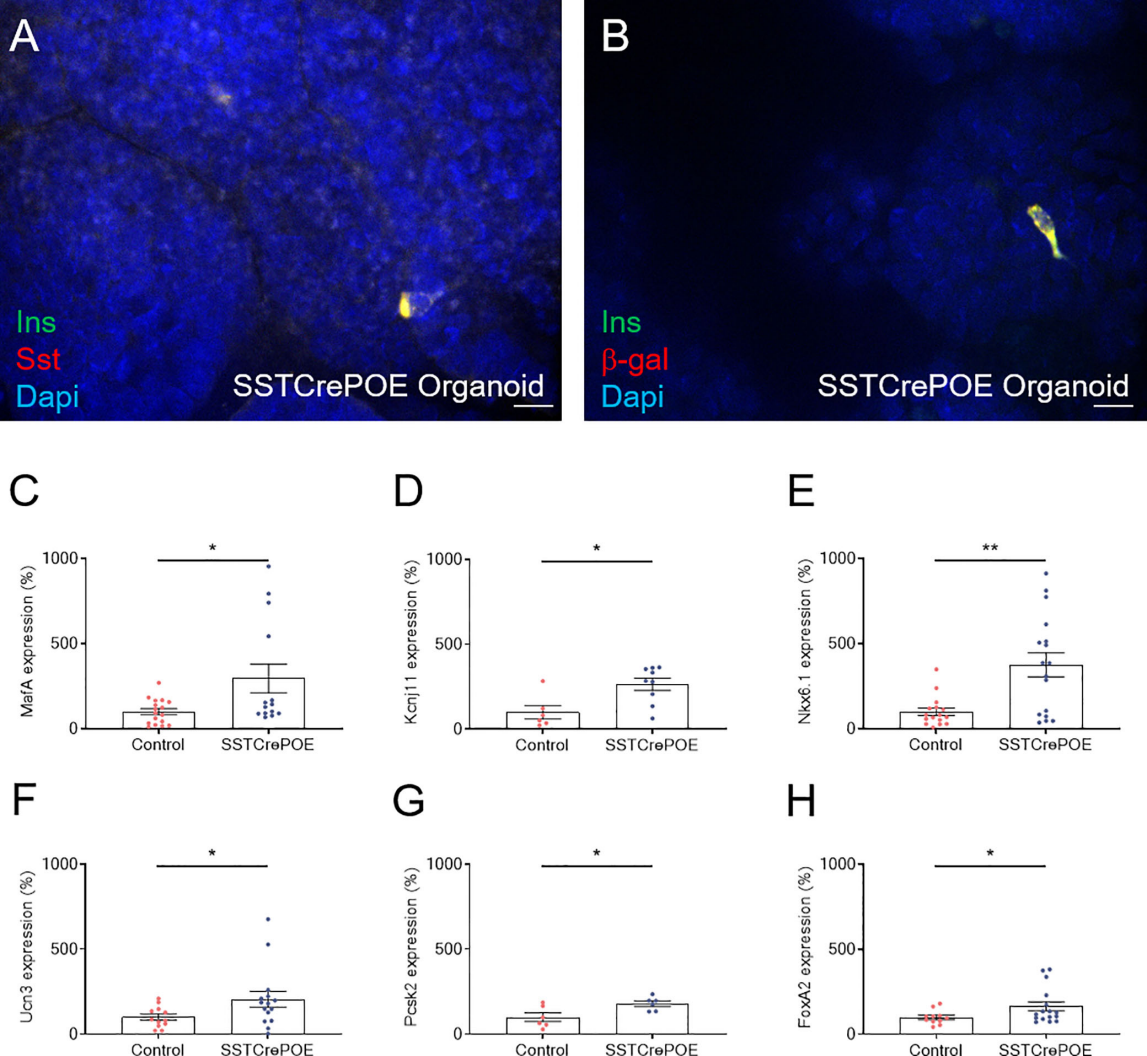
*Ex vivo* model characterisation. **(A)** Representative photograph of cells co-expressing insulin and somatostatin. **(B)** Representative photograph of transgenic gut organoids stained for insulin and β-galactosidase. Scale bar, 20μm. All insulin-producing cells are positive for the β-galactosidase lineage tracer, demonstrating a conversion of D cells into insulin-expressing cells. **(C–H)** Comparison of gene expression levels between control (red) and transgenic (blue) gut organoids focusing on beta-cell marker genes: *MafA* **(C)**, *Kcnj11* encoding for Kir6.2 **(D)**, *Nkx6.1* **(E)**, *Ucn3* **(F)**, *Pcsk2* encoding for PC2 **(G)** and *FoxA2* **(H)** (6 < n < 17). All beta-cell markers were found considerably increased at the expression level when comparing intestinal organoids derived from SSTCrePOE mice versus controls. *P < 0.05, **P < 0.01.

As seen *in vivo* at the protein level, we confirmed that somatostatin-expressing cells were found in all gut organoids analysed (**Figures 8A, C**). Importantly, insulin^+^ cells were exclusively detected in organoids derived from SSTCrePOE transgenic mice (**Figures 8B, D**). *Somatostatin* transcript levels were found 38% decreased in gut organoids derived from SSTCrePOE mice as compared to controls (**Figure 8E**). Importantly, *insulin* expression levels appeared 256% increased (and up to 805% augmented in some organoids) when comparing control and transgenic organoids (**Figure 8F**), again mimicking the results obtained *in vivo*. Another validation of such an *ex vivo* model came from the detection of insulin-labelled cells co-stained with somatostatin, again replicating *in vivo* observations where 65% of cells were found to express both hormones (**Figure 9A**). Furthermore, and most importantly, lineage-tracing experiments proved that cells positive for insulin were also labelled with β-galactosidase (**Figure 9B**). In a continued effort to determine whether gut organoids could faithfully mimic the *in vivo* mouse model, we assessed gene expression levels of key beta-cell markers (**Figures 9C–H**). A clear increase in the expression levels of *MafA*, *Kcnj11* (encoding for Kir6.2)*, Nkx6.1, Ucn3, Pcsk2* (encoding for PC2) and *FoxA2* was outlined (up to 275%), thus confirming *in vivo* data. Altogether, being the results obtained using gut organoids in agreement with the *in vivo* data, we could validate the use of this *ex vivo* approach as a model to explore the functional outcome of gastrointestinal beta-like cells.

### Gastrointestinal Beta-Like Cells Can Release Insulin Upon Stimulation

**Figure 10:**
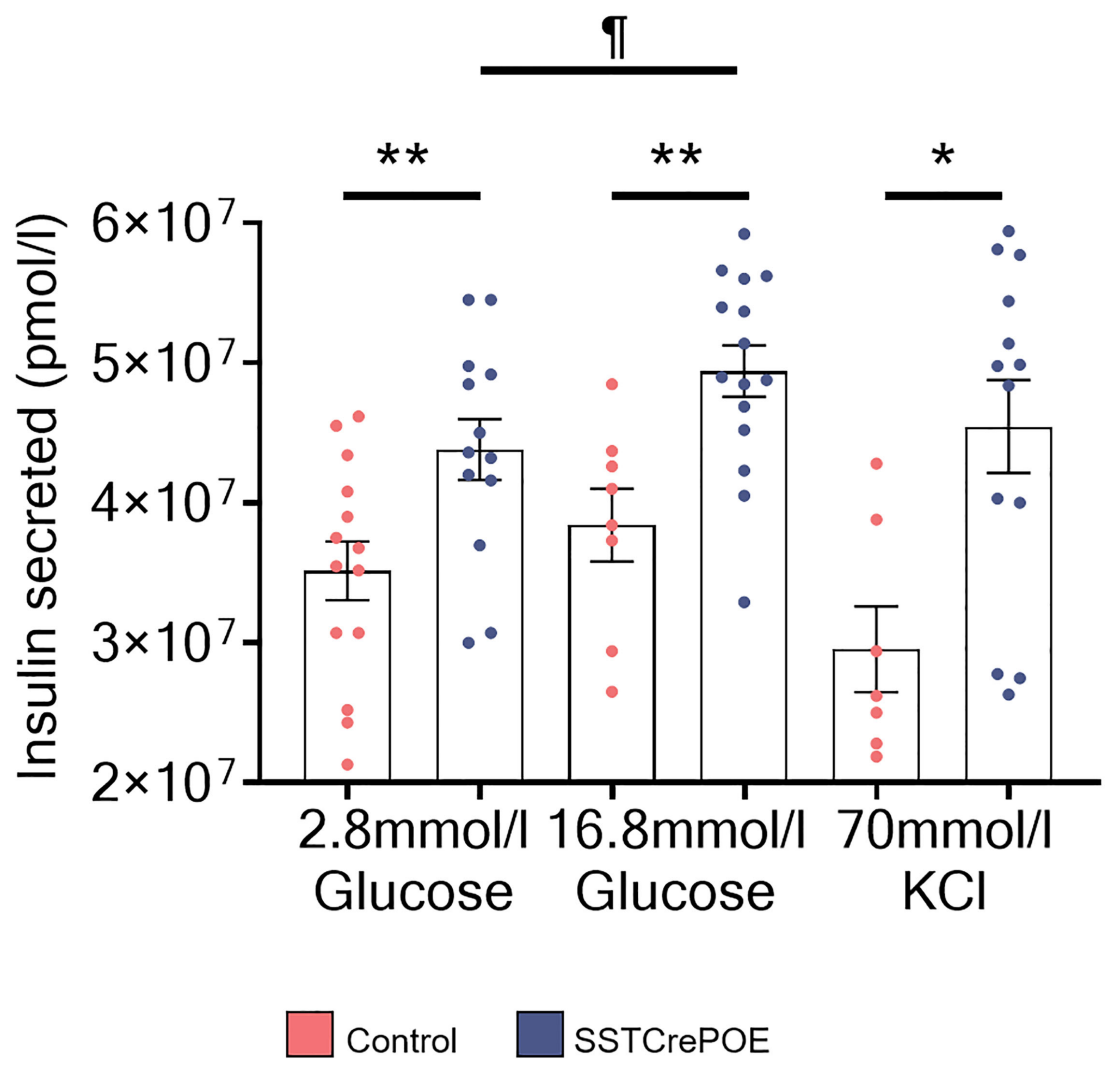
Regulated insulin secretion in SSTCrePOE gut organoids. Quantification of insulin amounts released from control (red) and SSTCrePOE (blue) gut organoids upon stimulation (7 < n < 15) with 2.8mM glucose, 16.8mM glucose and 70mM KCl. At higher concentration of glucose (16.8mM), transgenic intestinal organoids release significant higher amounts of insulin when compared to control and to SSTCrePOE organoids stimulated with lower concentration of glucose (2.8mM), suggesting that insulin from intestinal beta-like cells can be secreted in a regulated manner. The KCl stimulation acts as a positive control of the test, as KCl induces the exocytosis of the insulin vesicles. ^¶^P < 0.05, *P < 0.05, **P < 0.01.

Once the gut organoids showed their ability to replicate *in vivo* data, we wondered whether the neo-generated beta-like cells were functional. Therefore, glucose-stimulated insulin secretion (GSIS) analyses were performed. The medium from control and transgenic organoids was collected and the amount of insulin contained in the medium was analysed by ELISA (**Figure 10**). Interestingly, transgenic organoids released more insulin in the medium at basal glucose stimulation (2.8mM) when compared to control organoids (depleted of beta-cells). Equally important was the finding that the same organoids, when stimulated with a high glucose concentration (16.8mM), were able to secrete significantly higher amounts of insulin than with basal stimulation, suggestive of the ability of gastrointestinal beta-like cells to sense glucose and respond by secreting insulin in a regulated manner. As a last proof, organoids were challenged with KCl, which induces membrane depolarisation and, subsequently, an exocytosis of the secretory vesicles containing insulin. Yet again, a significantly higher insulin content was detected in the medium of SSTCrePOE organoids when compared to controls.

Together, these results clearly demonstrate that SSTCrePOE-derived organoids can release insulin upon glucose stimulation and that such secretion is regulated depending on the glucose concentration.

## Discussion

In the present study, we demonstrate that the misexpression of *Pax4* in gastrointestinal D cells triggers their conversion into insulin-expressing cells. These cells display a beta-like cell phenotype and can sense and respond to glucose by releasing insulin as demonstrated in an *ex vivo* environment. Our work thus demonstrates that *Pax4* gene modulation could represent an interesting path for reprogramming intestinal cells into beta-like cells, a result of great potential for diabetes research.

Somatostatin originating from the gastrointestinal tract has been reported to play an inhibitory role on other hormones secretion as well as to control gut motility and absorption rate (47, 53–57). Somatostatin-producing D cells are found scattered throughout the gastrointestinal mucosa, gastric D cells representing the major source of this hormone (44, 48, 58). Accordingly, we confirmed the greatest concentration in D cells in the stomach and, to a lesser extent, in the duodenum. Interestingly, the misexpression of *Pax4* was found sufficient to induce a dramatic decrease in D cells and consequently in somatostatin expression, in both tissues. However, few D cells could still be detected while circulating somatostatin levels were only reduced by 35%, indicating that the remaining D cells were able to compensate for their lost counterparts. This could be easily explained by the continued regeneration of the gastrointestinal tissue, and thus of D cells.

**Figure 11:**
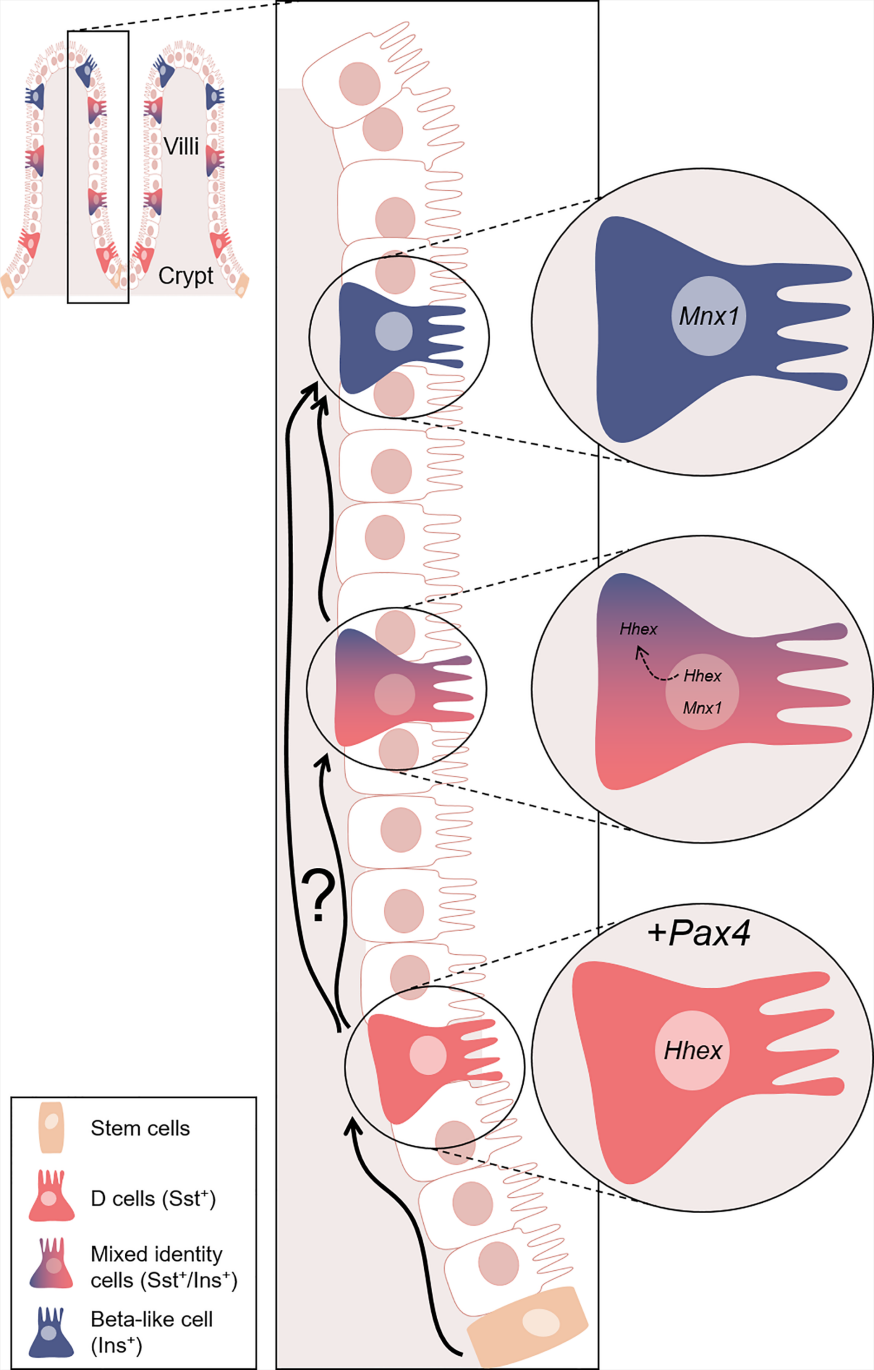
Model proposed for the *Pax4*-mediated conversion of D cells into beta-like cells. Representation of the gastrointestinal structure. In the crypts, tissue stem cells (orange) continuously (re)generate the intestinal epithelium, some of which eventually differentiating into D cells (red). In our model, D cells are genetically modified to ectopically express *Pax4*. This genetic alteration triggers the conversion of D cells into beta-like cells (blue). We hypothesised that the *Pax4*-mediated conversion occurs through the inactivation of *Hhex*, directly repressed by *Pax4* and/or indirect modulated through *Mnx1*. Interestingly, this conversion appears to be progressive, as supported by the observation of cells displaying a mixed identity. Hhex is expressed exclusively in the nucleus of D cells. However, Hhex was found in the nucleus and translocated in the cytoplasm of insulin^+^ cells, while the expression of *Mnx1* was increasing. At the last step of this conversion, insulin^+^ cells only express beta-cell markers. Nevertheless, one cannot exclude the possibility that some D cells are directly converted into beta-like cell without passing through a transitory bi-hormonal phase. Equally important is the observation that D cells lost to conversion are continuously replaced and yet again turned into beta-like cells.

Taking in consideration previous results demonstrating that, in the pancreas, the misexpression of *Pax4* in somatostatin-expressing delta-cells was associated with their conversion into beta-like cells (41), we wondered whether such conversion was similarly occurring in the gastrointestinal tissues. At the protein level multiple insulin^+^ cells were detected in stomach and duodenum of SSTCrePOE, both tissues being normally devoid of insulin-producing cells. Supporting this observation, *insulin* expression levels were found to be increased by up to 495% depending on the (st)age analysed in both duodenum and stomach transgenic samples. Lineage tracing experiments confirmed that all insulin-expressing cells were labelled with β-galactosidase (more than 100 cells examined), demonstrating that the sole misexpression of *Pax4* can turn D cells into insulin-producing cells. This was further confirmed by the detection of cells co-expressing both insulin and somatostatin. Of note, such detection of cells displaying a mixed identity suggest an initial slow and gradual process of conversion eventually resulting in single-hormone insulin-expressing cells (**Figure 11**).

Aiming to determine whether these insulin-expressing cells displayed a true beta-cell phenotype, we investigated the expression of *bona fide* beta-cell markers (59). At both the transcript and protein levels, the data obtained was of interest as we could prove a parallel increase (up to 480%) between the insulin^+^ cell counts and the levels of expression of key genes involved in defining the beta-cell identity (*MafA, Kcnj11, Nkx6.1, Ucn3, Pcsk2, FoxA2* and *Mnx1*), these being normally not expressed in the gastrointestinal tract of wild-type animals. Moreover, at the protein level, the observation of insulin^+^ cells co-labelled with C-peptide was also equally interesting, as it suggested that insulin went through all the classical maturation steps prior to initiating C-peptide expression. We also investigated the expression of PC1/3, an enzyme involved in the maturation of insulin itself: PC1/3 was thus clearly detected in insulin^+^ cells, a result demonstrating the normal post-translational processing of insulin. Similarly, the co-labelling of insulin^+^ cells with Kir6.2, which is the major subunit of the K_ATP_ channel involved in insulin secretion, indicated that the insulin^+^ cells could potentially release the hormone.

Interestingly and as previously discussed, such *Pax4*-mediated D-to-beta-like conversion appeared to be progressive, as outlined by the detection of cells displaying a mixed identity (**Figure 11**). This is further supported when focusing on the D cell marker, Hhex, a key transcription factor for their specification (60). As expected, Hhex was detected (1) in the nuclei of D cells, but also (2) in the nucleus or cytoplasm of a subpopulation of insulin^+^ cells, suggestive of D-to-beta-like transition phase. A last subpopulation (3) of insulin^+^ cells was outlined, these being depleted of Hhex, highlighting the completion of the identity shift.

One may therefore envision two hypotheses concerning the mechanisms underlying the *Pax4*-mediated D-to-beta-like cell conversion. In the first, Pax4 would directly inhibit *Hhex* expression. Accordingly, Pax4 binding sites have been detected within the *Hhex* locus at the position chr10:94451176-94451184 (61, 62)), which could explain the reduction of *Hhex* expression and the loss of the D cell identity. The other alternative would be a putative role of *Pax4* in modulating *Mnx1* expression. In previous reports, the repression of *Mnx1* was indeed shown to trigger pancreatic beta-to-delta-cell conversion (52). Accordingly, pancreata from animals lacking *Pax4* were found depleted of *Mnx1* expression, suggesting that Mnx1 might act downstream of Pax4 in β-cell determination (39). Hence, the increased *Mnx1* expression observed in SSTCrePOE gastrointestinal samples could underly the reverse conversion, that is from somatostatin-producing cells to insulin-expressing cells. Interestingly, according to the JASPAR database, *Mnx1* is predicted to regulate *Hhex*, which could suggest a putative indirect role of *Pax4* in *Hhex* inhibition. Obviously, additional studdies and alternative analyses focusing on D cell markers would be necessary to confirm these results but, for the latter, none of those tried did work in our hands when assayed by immunohistochemistry.

Focusing further on the mechanisms underlying the D-to-beta-like conversion observed in SSTCrePOE animals, one may wonder whether this process involved a dedifferentiation step prior to a re-differentiation event. To address this question, we assayed the expression of the endocrine precursor cell marker *Ngn3* (data not shown). No change in *Ngn3* expression or Ngn3-content was noted. One may thus conclude that the *Pax4*-mediated D-to-beta-like conversion most likely occurs through direct conversion (or *via* a limited de-differentiation/re-differentiation). Nevertheless, additional work would be required to gain further insight into the molecular mechanisms involved.

Considering the fact that the delta-to-beta-like cell conversion previously observed in SSTCrePOE pancreas prevented us from assessing the metabolic changes exclusively provoked by the gastrointestinal D-to-beta-like cell conversion, an *ex vivo* approach was preferred. We thus developed gut-derived mouse organoids to evaluate the functionality of the neo-generated gastrointestinal insulin^+^ cells. Importantly, intestinal beta-like cells appeared able to release insulin in a glucose dependent manner. Hence, from this work combined to thorough qPCR analyses focusing on beta-cell markers, we can conclude that the insulin^+^ cells detected in the gastrointestinal tract of SSTCrePOE transgenic mice display most beta-cell features and are functional.

The gastrointestinal tract harbours a great potential for T1D research due to its continuously regenerating nature. This feature is of great interest in the context of type 1 diabetes as reprogramming/engineering the gastrointestinal epithelium to generate insulin^+^ cells could allow for their continued replenishment despite a putative autoimmune attack. Here, we reported the conversion of D cells into functional beta-like cells upon the misexpression of *Pax4*. It is worth considering that the neo-generated gastrointestinal insulin-producing cells are (1) directly exposed to the ingested food, (2) continuously replaced, and (3) scattered in an immune-tolerogenic environment, which altogether renders the aforementioned conversion of great interest in the context of type 1 diabetes research. However, prior to considering such translational approach, some limitations need to be addressed. Indeed, (1) determining whether human D cells harbour an equivalent Pax4-mediated plasticity when compared to their mouse counterparts would be required, despite reports suggesting comparable transcriptomes and peptidomes (63). Similarly, (2) understanding whether the neo-generated gastrointestinal insulin-expressing cells would escape the autoimmune attack and/or promote systemic immune tolerance would need to be investigated. Altogether, this work suggests that identifying *Pax4* targets, and putative druggable targets, would be of great interest to gain further insight into the molecular mechanisms involved in this *Pax4*-mediated conversion, but also for the development of therapies aiming at regenerating the endogenous beta-cell mass.

## Data Availability Statement

The raw data supporting the conclusions of this article will be made available by the authors, without undue reservation.

## Ethics Statement

The animal study was reviewed and approved by Ciepal-Azur.

## Author Contributions

AG-U, ND, and PC conceived, designed the experiments, and discussed the results. AG-U performed most of the experiments, acquired the data and analysed/interpreted the results. CA, MF, SB, TN, and SS supported designing and performing some experiments. JA contributed to data acquisition. AG-U drafted and wrote the manuscript. AG-U, CA, MF, JA, SB, TN, SS, ND, and PC revised the manuscript and approved the final version. PC is the guarantor of this work. All authors contributed to the article and approved the submitted version.

## Funding

The authors are supported by the JDRF (2-SRA-2017-416-S-B, 2-SRA-2017-417-S-B), the Agence Nationale pour la Recherche (ANR-16-CE18-0005-01, ANR-17-CE14-0034), MSD-Avenir, and French Government (National Research Agency, ANR) through the “Investments for the Future” programs LABEX SIGNALIFE ANR-11-LABX-0028-01 and IDEX UCAJedi ANR-15-IDEX-01.

## Conflict of Interest

Author SB was employed by PlantaCorp GmbH.

The remaining authors declare that the research was conducted in the absence of any commercial or financial relationships that could be construed as a potential conflict of interest.

## Publisher’s Note

All claims expressed in this article are solely those of the authors and do not necessarily represent those of their affiliated organizations, or those of the publisher, the editors and the reviewers. Any product that may be evaluated in this article, or claim that may be made by its manufacturer, is not guaranteed or endorsed by the publisher.

## Supplementary Material

Supplementary Figure 1 Evaluation of somatostatin, IL-1beta and TNF-alpha circulating blood levels in SSTCrePOE mice. Retroorbital blood extraction was performed in 150-day-old SSTCrePOE and Control mice. **(A)** Somatostatin circulating levels appeared reduced in SSTCrePOE animals (n=4) when compared to controls (n=6), confirming the reduction in somatostatin displayed by this phenotype. **(B, C)** Assessment of proinflammatory cytokines blood levels. IL-1beta levels were found unaltered in SSTCrePOE animals (n=7) when comparing with control samples (n=6) **(B)**. Similarly, no differences were detected in TNF-alpha circulating levels when comparing blood samples from controls (n=7) and transgenic animals (n=7) **(C)**, indicative of an unaltered gastrointestinal barrier in SSTCrePOE animals.

Supplementary Figure 2 Immunohistochemical characterisation of SSTCrePOE GI tract. Representative photograph of duodenum **(A–G)** and stomach **(H, I)** sections from SSTCrePOE animals stained for insulin (green) and markers of different cell populations (red). **(A)** Epcam, an epithelial cell marker also labels insulin^+^ cells. **(B)** Muc2, a marker of Goblet cells outliones two different populations: insulin^+^ cells and Goblet cells. **(C)** Paneth cells staining using anti-Lyz antibodies. Insulin^+^ cells are clearly distinguished from the Paneth cell population. **(D)** Dcamkl1 recognises stem cells. Tissue stem cells are thus found located away from insulin^+^ cells. **(E)** ChgA labels enteroendocrine cells. Note that insulin^+^ cells are co-stained with ChgA while ChgA^+^/Ins^-^ cells are expectedly also detected. **(F–I)** Among the EEC population, L cells are marked by GLP1**(F)**, K cells by GIP **(G, H)**, and G cells by Gas **(I)**: importantly, none of these express insulin. Scale bar, 20μm, insets 5μm.

Supplementary Figure 3 SSTCrePOE animals display an improved glucose tolerance. **(A)** SSTCrePOE (n=13) (blue) and control (n=9) (red) mice were subjected to an ipGTT. SSTCrePOE animals performed better than controls with a lower peak in glycemia and a faster return to normoglycemia. This suggests an increase in the functional beta-cell fraction. **(B)** Representation of the ipGTT data as area under the curve (AUC). **(C)** SSTCrePOE (n=13) (blue) and control (n=9) (red) mice were subjected to an oGTT. A small trend (albeit non-significant) towards an improved oral glucose tolerance in transgenic mice versus controls was outlined. **(D)** Representation of the oGTT data as area under the curve (AUC).
