# Supplementary figures and images for: Conversion of Gastrointestinal Somatostatin-Expressing D Cells Into Insulin-Producing Beta-Like Cells Upon Pax4 Misexpression
Source: Front Endocrinol (Lausanne). 2022 Apr 29;13:861922. doi: 10.3389/fendo.2022.861922 (PMC9103212; doi:10.3389/fendo.2022.861922)

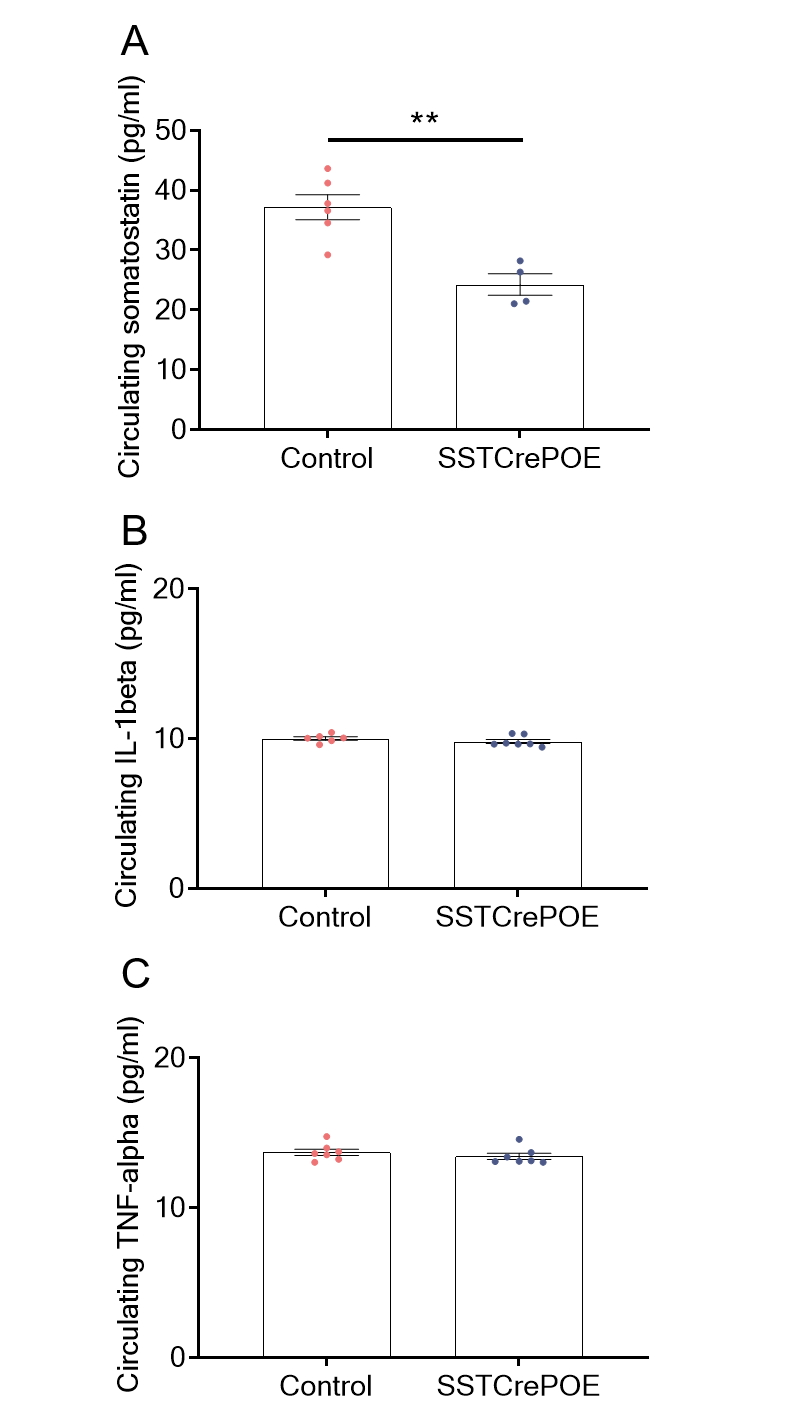

Supplement: Supplementary Figure 1 — Evaluation of somatostatin, IL-1beta and TNF-alpha circulating blood levels in SSTCrePOE mice. Retroorbital blood extraction was performed in 150-day-old SSTCrePOE and Control mice. (A) Somatostatin circulating levels appeared reduced in SSTCrePOE animals (n=4) when compared to controls (n=6), confirming the reduction in somatostatin displayed by this phenotype. (B, C) Assessment of proinflammatory cytokines blood levels. IL-1beta levels were found unaltered in SSTCrePOE animals (n=7) when comparing with control samples (n=6) (B). Similarly, no differences were detected in TNF-alpha circulating levels when comparing blood samples from controls (n=7) and transgenic animals (n=7) (C), indicative of an unaltered gastrointestinal barrier in SSTCrePOE animals. [file Image_1.tif]

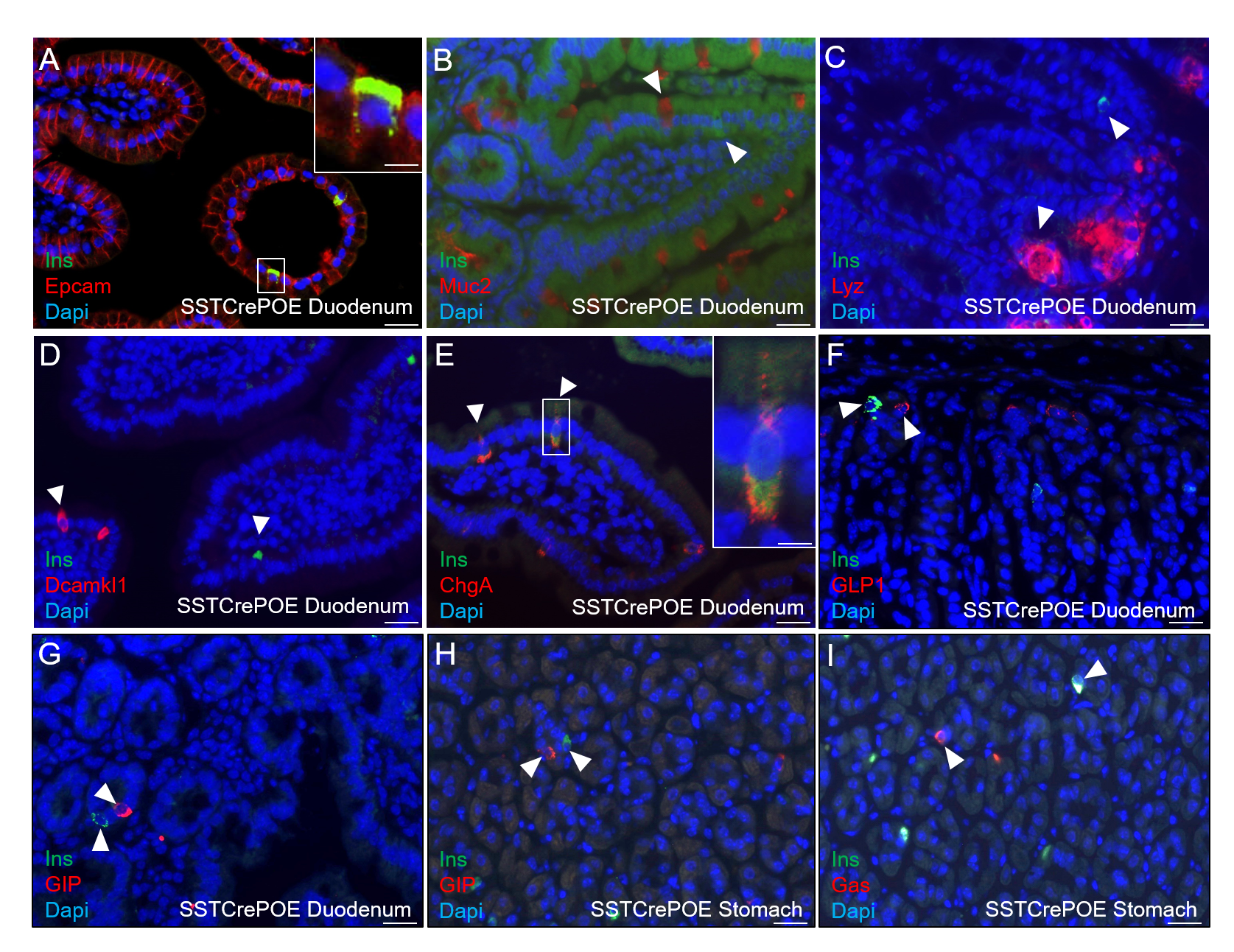

Supplement: Supplementary Figure 2 — Immunohistochemical characterisation of SSTCrePOE GI tract. Representative photograph of duodenum (A–G) and stomach (H, I) sections from SSTCrePOE animals stained for insulin (green) and markers of different cell populations (red). (A) Epcam, an epithelial cell marker also labels insulin+ cells. (B) Muc2, a marker of Goblet cells outliones two different populations: insulin+ cells and Goblet cells. (C) Paneth cells staining using anti-Lyz antibodies. Insulin+ cells are clearly distinguished from the Paneth cell population. (D) Dcamkl1 recognises stem cells. Tissue stem cells are thus found located away from insulin+ cells. (E) ChgA labels enteroendocrine cells. Note that insulin+ cells are co-stained with ChgA while ChgA+/Ins- cells are expectedly also detected. (F–I) Among the EEC population, L cells are marked by GLP1(F), K cells by GIP (G, H), and G cells by Gas (I): importantly, none of these express insulin. Scale bar, 20μm, insets 5μm. [file Image_2.tif]

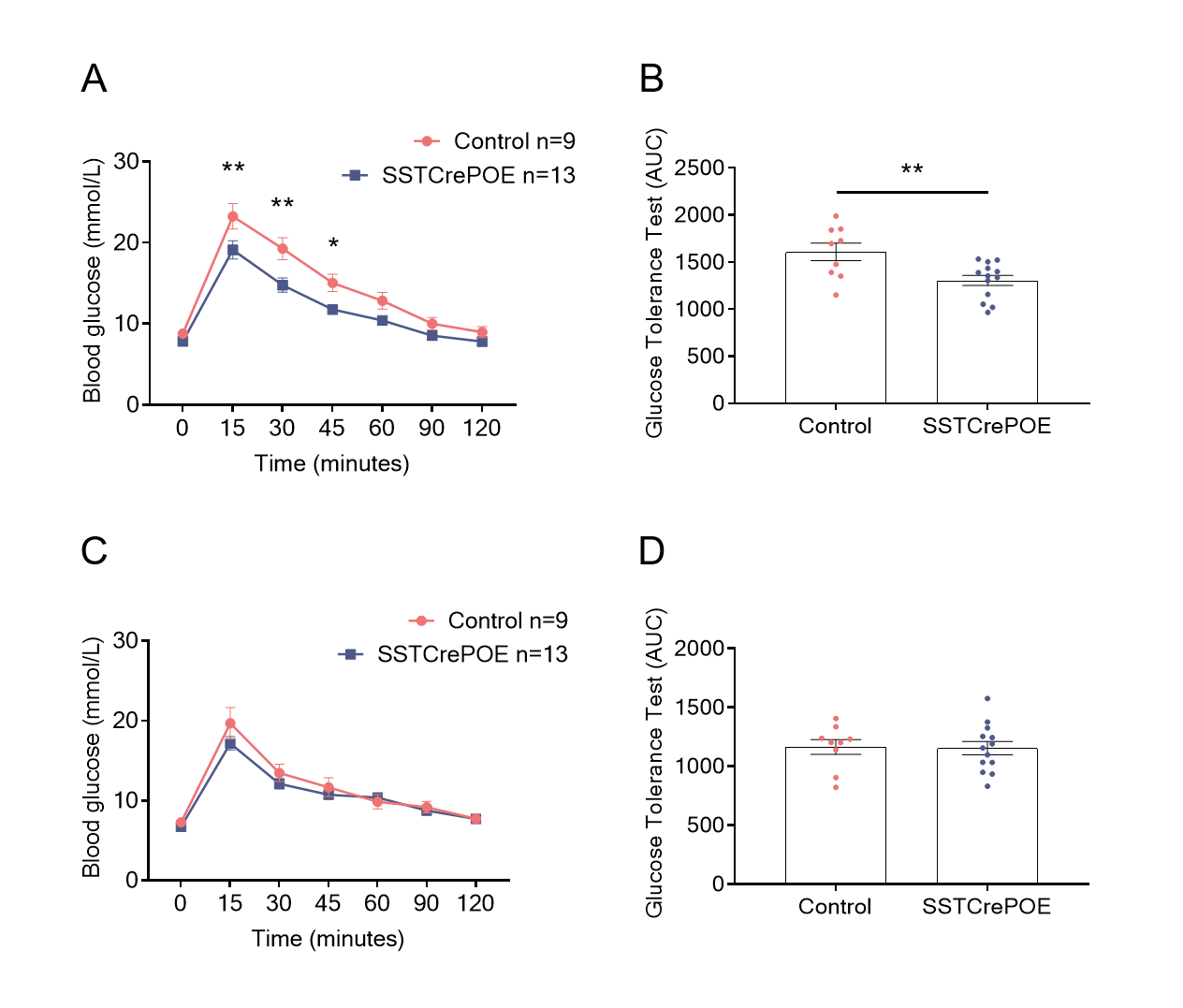

Supplement: Supplementary Figure 3 — SSTCrePOE animals display an improved glucose tolerance. (A) SSTCrePOE (n=13) (blue) and control (n=9) (red) mice were subjected to an ipGTT. SSTCrePOE animals performed better than controls with a lower peak in glycemia and a faster return to normoglycemia. This suggests an increase in the functional beta-cell fraction. (B) Representation of the ipGTT data as area under the curve (AUC). (C) SSTCrePOE (n=13) (blue) and control (n=9) (red) mice were subjected to an oGTT. A small trend (albeit non-significant) towards an improved oral glucose tolerance in transgenic mice versus controls was outlined. (D) Representation of the oGTT data as area under the curve (AUC). [file Image_3.tif]
